# SPARC in cancer‐associated fibroblasts is an independent poor prognostic factor in non‐metastatic triple‐negative breast cancer and exhibits pro‐tumor activity

**DOI:** 10.1002/ijc.34345

**Published:** 2022-11-30

**Authors:** Lindsay B. Alcaraz, Aude Mallavialle, Caroline Mollevi, Florence Boissière‐Michot, Hanane Mansouri, Joelle Simony‐Lafontaine, Valérie Laurent‐Matha, Thierry Chardès, William Jacot, Andrei Turtoi, Pascal Roger, Séverine Guiu, Emmanuelle Liaudet‐Coopman

**Affiliations:** ^1^ IRCM, INSERM U1194 Univ Montpellier, ICM Montpellier France; ^2^ Biometry Unit, ICM University of Montpellier Montpellier France; ^3^ Desbrest Institute of Epidemiology and Public Health University of Montpellier, INSERM Montpellier France; ^4^ Translational Research Unit, ICM Montpellier France; ^5^ RHEM, IRCM Montpellier France; ^6^ Department of Medical Oncology ICM Montpellier France; ^7^ Department of Pathology CHU Nîmes France

**Keywords:** CAF, osteonectin, single‐cell mRNA sequencing, SPARC, TNBC

## Abstract

Triple‐negative breast cancer (TNBC) is the most aggressive breast cancer subtype and lacks specific targeted therapeutic agents. The current mechanistic evidence from cell‐based studies suggests that the matricellular protein SPARC has a tumor‐promoting role in TNBC; however, data on the clinical relevance of SPARC expression/secretion by tumor and stromal cells in TNBC are limited. Here, we analyzed by immunohistochemistry the prognostic value of tumor and stromal cell SPARC expression in 148 patients with non‐metastatic TNBC and long follow‐up (median: 5.4 years). We also quantified PD‐L1 and PD‐1 expression. We detected SPARC expression in tumor cells (42.4%), cancer‐associated fibroblasts (CAFs; 88.1%), tumor‐associated macrophages (77.1%), endothelial cells (75.2%) and tumor‐infiltrating lymphocytes (9.8%). Recurrence‐free survival was significantly lower in patients with SPARC‐expressing CAFs. Multivariate analysis showed that SPARC expression in CAFs was an independent prognostic factor. We also detected tumor and stromal cell SPARC expression in TNBC cytosols, and in patient‐derived xenografts and cell lines. Furthermore, we analyzed publicly available single‐cell mRNA sequencing data and found that in TNBC, *SPARC* is expressed by different CAF subpopulations, including myofibroblasts and inflammatory fibroblasts that are involved in tumor‐related processes. We then showed that fibroblast‐secreted SPARC had a tumor‐promoting role by inhibiting TNBC cell adhesion and stimulating their motility and invasiveness. Overall, our study demonstrates that SPARC expression in CAFs is an independent prognostic marker of poor outcome in TNBC. Patients with SPARC‐expressing CAFs could be eligible for anti‐SPARC targeted therapy.

## Graphical abstract

What's new?

In vitro evidence suggests that the matricellular protein SPARC has a tumor‐promoting role in triple‐negative breast cancer (TNBC). However, the clinical relevance of SPARC in triple‐negative breast cancer remains unclear. Here, the authors analyzed the prognostic value of tumor and stromal SPARC in 148 patients with non‐metastatic TNBC. SPARC was most often expressed by myofibroblasts and inflammatory cancer‐associated fibroblasts (CAFs), and fibroblast‐secreted SPARC exhibited a tumor‐promoting role in TNBC. Moreover, SPARC expression in CAFs was an independent prognostic marker of poor outcome. This study points to CAF‐derived SPARC as a potential novel therapeutic target in triple‐negative breast cancer.

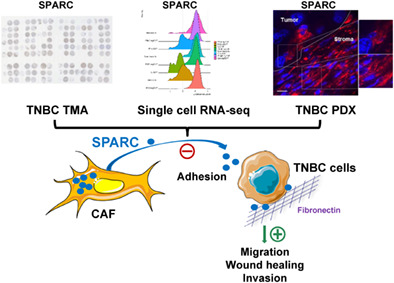

## 1 INTRODUCTION

Triple‐negative breast cancers (TNBC) are defined by the lack of estrogen receptor (ER), progesterone receptor (PR) and HER2 expression/amplification. TNBC represent 15% of all breast cancers.^1^ Despite surgery, adjuvant chemotherapy and radiotherapy, TNBC prognosis is poor, mainly due to the disease heterogeneity and lack of specific therapeutic targets. TNBC is characterized by its unique tumor microenvironment that differs from that of other breast cancer subtypes and promotes cancer cell proliferation, angiogenesis and drug resistance, while inhibiting apoptosis and tumor immune suppression.^2^ TNBC microenvironment components, such as transformed extracellular matrix, soluble factors, immune cells and re‐programmed fibroblasts, hamper the host antitumor response and helps tumor progression and metastasis formation. In TNBC, stroma heterogeneity remains poorly understood, thus limiting the development of stromal cell‐targeted therapies.

In the tumor microenvironment, heterogeneous populations of fibroblast‐like cells, collectively termed cancer‐associated fibroblasts (CAFs), are key players in the multicellular, stroma‐dependent alterations that contribute to cancer initiation and progression.^3^ However, not all CAFs are tumor supportive.^4^ For instance, normal fibroblasts have been shown to suppress tumor formation.^5^ In breast cancer, CAF abundance has been associated with aggressive adenocarcinomas and predicts disease recurrence.^6^, ^7^ In TNBC, recent single‐cell RNA sequencing (scRNA‐seq) studies highlighted a considerable CAF heterogeneity. The CAF subpopulations that contribute to immune suppression, inflammation and chemoresistance are now increasingly better characterized.^8^, ^9^, ^10^, ^11^ In breast cancer, tumor‐associated macrophages (TAMs) are the most abundant inflammatory cells, and are typically M2‐polarized cells with suppressive capacity^12^ linked to their enzymatic activities and anti‐inflammatory cytokine production.^13^ TAMs support tumor progression and metastasis formation by blocking the anti‐tumor immunity and by secreting factors that promote angiogenesis and epithelial‐to‐mesenchymal transition.^12^ High M2‐polarized TAM levels are associated with poorer TNBC outcome.^14^ Tumor‐infiltrating lymphocytes (TILs) constitutes a robust and independent prognostic marker in TNBC treated with (neo)adjuvant chemotherapy.^15^, ^16^ TILs are associated with improved disease‐free and overall survival (OS) rates in TNBC.^17^ Programmed cell death (PD‐1) (a CD‐28‐CTLA‐4 family member) is an immune check‐point receptor expressed by immune cells that contributes to the immune tolerance of self‐antigens by peripheral T cells. PD‐L1 (one of its ligand) is expressed by immune cells, epithelial breast cancer cells and TILs. Activation of the PD‐1‐PD‐L1 pathway specifically inhibits T‐cell activation, and is one of the mechanisms that allow cancer cells to escape the antitumor immune response.^18^ It is thought that TNBC are more immunogenic than other breast cancers. Indeed, the available evidence indicates that in TNBC, PD‐L1 expression is more frequent (up to 60%) than in other breast cancers, and that PD‐L1 tumor expression is positively associated with stromal TILs.^19^

The matricellular protein Secreted Protein Acidic and Rich in Cysteine (SPARC; also known as osteonectin or basement membrane 40, BM40) is a Ca^2+^‐binding glycoprotein that regulates extracellular matrix assembly and deposition, growth factor signaling and cell‐stroma interactions.^20^, ^21^, ^22^, ^23^ In cancer, SPARC is mainly secreted by neighboring stromal cells, and to a lower extent by tumor cells.^24^, ^25^, ^26^ SPARC plays oncogenic or tumor‐suppressive roles, depending on the cancer type.^27^, ^28^ In breast cancer, SPARC has been associated with worse prognosis and has pro‐tumor functions.^25^, ^29^, ^30^, ^31^, ^32^, ^33^, ^34^ In TNBC cells, SPARC stimulates their migration and invasion,^35^ and promotes MMP‐2 activation, thereby contributing to the proteolytic cascades associated with tumor invasion.^36^ Moreover, SPARC stimulates tumor growth and lung colonization after grafting of mouse 4T1 and LM3 TNBC cells in syngeneic mice by promoting cell cycling and expansion of myeloid‐derived suppressor cells.^33^ Conversely, SPARC transfection in high‐grade isogenic breast cancer cells reduces tumor rate, and favors epithelial‐to‐mesenchymal transition and the formation of a highly immunosuppressive microenvironment composed of immune cells, such as myeloid‐derived suppressor cells.^31^ Some in vitro studies in which SPARC was overexpressed or silenced in cancer cells showed its inhibitory effect on cancer cell motility, invasion and proliferation.^37^, ^38^, ^39^ In TNBC, mechanistic cell‐based studies support a tumor‐promoting role,^40^ suggesting that SPARC could be a candidate stromal therapeutic target.

The aim of this study was to evaluate SPARC expression in tumor and stromal cells, their prognostic value, and correlation with fibrosis, TAM infiltration, TIL density, PD‐L1 and PD‐1 levels in a large series of patients with non‐metastatic TNBC. The objective was to identify a TNBC subgroup with worse prognosis and eligible for stroma‐targeted therapy focused on extracellular matrix proteins.

## 2 MATERIALS AND METHODS

### 2.1 Antibodies and reagents

The rabbit polyclonal anti‐SPARC (15274‐1‐AP) and the mouse monoclonal anti‐periostin (clone No 1A11A3) antibodies were purchased from Proteintech. The mouse monoclonal anti‐SPARC (clone AON‐5031, sc‐73 472) and the mouse monoclonal anti‐HSC70 (clone B‐6, sc‐7298) antibodies were purchased from Santa Cruz Biotechnology. The mouse monoclonal anti‐tubulin antibody (clone 236‐10 501, #A11126) was from Thermo Fisher Scientific. The mouse monoclonal anti‐cytokeratin 5/6 antibody (clone 6D5/16 B4) was from Dako. The mouse monoclonal anti‐epidermal growth factor receptor (EGFR) antibody (clone 31G7) was from inVitroGen. The mouse monoclonal anti‐PD‐1 (clone MRQ‐22), and the mouse monoclonal anti‐CD163 (clone 10D6) antibodies were from BioSB. The rabbit monoclonal anti‐PD‐L1 (clone SP142) was from Roche. The horse anti‐mouse immunoglobulin G (IgG)‐horseradish peroxidase (#7076), and goat anti‐rabbit IgG‐HRP (#7074 S) secondary antibodies were from Cell Signaling Technology. The donkey anti‐goat HRP conjugated antibody (FT‐1I7890) was from Interchim. The Alexa Fluor 488‐conjugated anti‐rabbit IgG (#Ab150077) was purchased from Abcam, and the Alexa Fluor 594‐conjugated anti‐mouse IgG (711‐585‐152) from ImmunoResearch Laboratories. Hoechst 33342 (#FP‐BB1340) was from Interchim FluoProbes.

### 2.2 Patients and tumor samples

TNBC tissue micro‐arrays (TMAs) included tissue samples from 148 patients with unifocal, unilateral, non‐metastatic TNBC who underwent surgery at Montpellier Cancer Institute between 2002 and 2012. All patients were informed before surgery that their surgical specimens may be used for research purposes. Patients did not receive neoadjuvant chemotherapy before surgery. ER and PR negativity were defined as <10% expression by immunohistochemistry (IHC), and HER2 negativity was defined as IHC 0/1+ or 2+ and negative by fluorescent/chromogenic hybridization in situ. The study approval for patient‐derived xenografts (PDXs) was previously published.^41^

### 2.3 Construction of TNBC TMAs

Tumor tissue blocks with enough material at gross inspection were selected from the Biological Resource Center. The presence of tumor tissue in sections was evaluated by a pathologist after hematoxylin‐eosin‐saffron (HES) staining of few sections. Two representative tumor areas were identified on each slide from which two malignant cores (1 mm in diameter) were extracted with a manual arraying instrument (Manual Tissue Arrayer 1, Beecher Instruments, Sun Prairie, WI, USA). After arraying completion, 4 μm sections were cut from the TMA blocks. One section was stained with HES and the others were used for IHC.

### 2.4 TMA IHC

TMA sections were incubated with antibodies against SPARC (clone AON‐5031), cytokeratin 5/6 (clone 6D5/16 B4), EGFR (clone 31G7), PD‐1 (clone MRQ‐22), PD‐L1 (clone SP142) and CD163 (clone 10D6) on a Autostainer Link48 platform (Dako) using the EnVision FLEX system (Dako) for signal amplification and diaminobenzidine tetrahydrochloride as chromogen. TMA sections were analyzed independently by two trained observers both blinded to the clinicopathological characteristics and patient outcomes. In case of disagreement, sections were revised by a third observer to reach a consensus. Results from duplicate cores, when available, were averaged. Basal‐like phenotype was defined by cytokeratin 5/6 and/or EGFR expression (>10% of tumor cells). SPARC signal in cancer cells was scored as negative (<1% of stained cells), or positive (≥ 1% of stained cells). SPARC signal in CAFs, TAMs, endothelial cells and TILs was scored as negative (<50% of stained cells), or positive (≥50% of stained cells). SPARC signal in normal epithelial breast tissue samples (N) was compared with the paired tumor sample (T) and scored as lower (N < T), equal (=), or higher (N ≥ T). TIL density (peritumoral and intratumoral) was evaluated on HE‐stained sections, and was scored as: 0 (no TILs), 1 (rare TILs), 2 (moderate infiltrate, fewer TILs than tumor cells) and 3 (diffuse infiltrate, more TILs than tumor cells). Fibrosis was evaluated on HE‐stained sections, and was scored as: 0 (no CAF), >20%, 20%‐50%, >50% of fibrosis. PD‐1 expression by TILs was scored as follows: not evaluable (no TILs), 0 (no stained TIL), 1 (<10% of stained TILs), 2 (10‐50% of stained TILs) and 3 (>50% of stained TILs). PD‐L1 expression in tumor cells was considered positive if detected in ≥1% of cells. TAM density was scored in CD163‐stained sections and compared with the TIL density: 0 (no TAM), 1 (rare TAMs), 2 (moderate infiltrate, fewer TAMs than TILs) and 3 (diffuse infiltrate, more TAMs than TILs).

### 2.5 Immunofluorescence analysis

Paraffin‐embedded PDX tissue sections were deparaffined, rehydrated, rinsed and saturated in PBS with 5% fetal calf serum (FCS) at 4°C overnight. Sections were incubated with 1.2 μg/ml anti‐SPARC rabbit polyclonal antibody (15274‐1‐AP) and 5 μg/ml anti‐periostin mouse monoclonal antibody (1A11A3), followed by incubation with AlexaFluor 488‐conjugated anti‐rabbit IgG and AlexaFluor 594‐conjugated anti‐mouse IgG (1/400), respectively. Nuclei were stained with 0.5 μg/ml Hoechst 33342. Sections were imaged with a 63× Plan‐Apochromat objective on z stacks with a Zeiss Axio Imager light microscope equipped with Apotome to eliminate out‐of‐focus fluorescence.

### 2.6 TNBC cytosols, cell lines, conditioned medium and western blotting

TNBC cytosols were previously prepared and frozen.^42^ The MDA‐MB‐453 (RRID:CVCL_0418), MDA‐MB‐436 (RRID:CVCL_0623), MDA‐MB‐468 (RRID:CVCL_0419), Hs578T (RRID:CVCL_0332), BT‐549 (RRID:CVCL_1092) and HCC1806 (RRID:CVCL_1258) TNBC cell lines were obtained from SIRIC Montpellier Cancer. The SUM159 (RRID:CVCL_5423) TNBC cell line was from Asterand (Bioscience, UK). The MDA‐MB‐231 (RRID:CVCL_0062), TNBC cell line was previously described.^43^ Human mammary fibroblasts (HMFs) were provided by J. Loncarek and J. Piette (CRCL Val d'Aurelle‐Paul Lamarque, Montpellier, France),^44^ THP1 (RRID:CVCL_0006) monocytes by L. Gros (IRCM, Montpellier), and primary human umbilical vein endothelial cells (HUVECs) by M. Villalba (IRMB, Montpellier). Cell lines were cultured in DMEM with 10% FCS (EuroBio), except the SUM159 cell line (RPMI with 10% FCS) and the THP1 cell line (RPMI with 10% decomplemented FCS, 10 mM HEPES, 1 mM sodium pyruvate and 50 μM β‐mercaptoethanol). THP1 monocytes were differentiated into M0 macrophages by exposure to phorbol 12‐myristate 13‐acetate (100 ng/ml; Sigma Aldrich) for 48 h. Then, cells became adherent and the medium was replaced with fresh medium supplemented with interleukin‐4 (20 ng/ml) for 24 h to induce differentiation of M0 macrophages to M2‐polarized macrophages. The M2‐polarized THP1 phenotype was validated by analyzing CD206 expression by RT‐qPCR (Supplementary Materials and Methods). All experiments were performed with mycoplasma‐free cells. All cell lines were authenticated by short tandem repeat profiling within the last 3 years of their use. For western blotting experiments, cell lysates were prepared in lysis buffer (50 mM HEPES [pH 7.5], 150 mM NaCl, 10% glycerol, 1% Triton X‐100, 1.5 mM MgCl_2_, 1 mM EGTA) containing cOmplete Protease Inhibitor Cocktail (Roche, Switzerland), and centrifuged at 13000×*g* for 10 min. The corresponding conditioned media were centrifuged at 500×*g* for 5 min. Proteins from whole cytosols (20 μg) or cell lysates (30 μg) and conditioned media (40 μl) were separated on 13.5% SDS‐PAGE and analyzed by immunoblotting with the anti‐SPARC (clone AON‐5031) and anti‐tubulin antibodies using standard techniques. To prepare conditioned medium, HMFs were grown to 90% confluence in DMEM complemented with 10% FCS. Following washes with phenol red‐ and serum‐free medium to remove serum proteins, cells were incubated in DMEM buffered with 50 mM HEPES [pH 7.5] and without FCS for 24 h. Medium was harvested, and centrifuged at 1000 rpm for 5 min, followed or not by SPARC depletion. Briefly, HMF conditioned medium was incubated with 5 μg of monoclonal anti‐human SPARC antibody (clone AON‐5031, sc‐73 472) overnight, and pre‐absorbed to protein G‐agarose at 4°C. Then conditioned medium (SPARC‐immunodepleted or not) was filtered using 0.22 μm filters to eliminate cell debris. Cleared HMF conditioned medium (HFM CM) was collected and added to MDA‐MB‐231 cells for in vitro functional assays. SPARC immunodepletion was confirmed by western blotting.

### 2.7 ScRNA‐seq data meta‐analysis

To evaluate SPARC expression in different cell subtypes, previously published scRNA‐seq data were used. The first study included five patients with TNBC,^9^ the second included six patients with TNBC,^10^ and the third included eight patients with luminal and TNBC tumors.^11^ Aligned 10× Genomics (Pleasanton, CA, USA) NGS data, obtained from the public archives (European Nucleotide Archive accession code PRJEB35405, Gene Expression Omnibus database accession code GSE118390 and European Genome‐Phenome Archive accession number AS00001004031), were loaded in R (4.0) and processed using the Seurat 3.4 package and default parameters.^45^ Individual cell populations were annotated as published in the original scRNA‐seq study^9^, ^10^, ^11^ with minor modifications when appropriate. To take into account CAF heterogeneity in the study by Karaayvaz et al,^10^ the clearly different CAF populations, which were merged in this previous analysis, were named CAF‐A, CAF‐B and CAF‐C.

### 2.8 Cell adhesion, migration and invasion assays

MDA‐MB‐231 cell adhesion was assessed as previously described.^40^ Briefly, 96‐well plates were coated with fibronectin (10 μg/ml; sc‐29 011; Santa Cruz Biotechnology) at 4°C overnight, and saturated with 1% bovine serum albumin (BSA) in PBS. MDA‐MB‐231 cells were detached with HyQTase (HyClone), washed in DMEM without FCS, and 5 10^4^ cells were then plated and incubated in serum‐free HMF CM (SPARC‐immunodepleted or not) at 37°C for 30 min. Non‐adherent cells were removed by flotation on a dense Percoll solution containing 3.33% NaCl (1.10 g/L), and adherent cells were fixed (10% [vol/vol] glutaraldehyde) using the buoyancy method.^46^ Cells were stained with 0.1% crystal violet, and absorbance was measured at 570 nm. For migration and invasion assays, 8‐μm pore Transwell inserts (polyvinyl pyrrolidone‐free polycarbonate filters) in 24‐well plates (Corning Inc., Corning, NY) were coated with 10 μg/ml fibronectin (500 ng) (migration assays) or Matrigel (100 μg, Corning) (invasion assays) at 4°C for 24 h. MDA‐MB‐231 cells were plated (5 × 10^4^ cells/well) in serum‐free HMF CM (SPARC‐immunodepleted or not) on the coated insert in the upper chamber. In these different assays, DMEM supplemented with 10% FCS was used as chemoattractant in the bottom chamber. After 16 h, non‐migrating/non‐invading cells on the apical side of each insert were scraped off with a cotton swab, and migration and invasion were analyzed with two methods: (1) migrating/invading cells were fixed in methanol, stained with 0.1% crystal violet for 30 min, rinsed in water, and imaged with an optical microscope. Two images of the pre‐set field per insert were captured (×100); (2) migrating/invading cells were incubated with 3‐(4,5‐dimethylthiazol‐2‐yl)‐2,5‐diphenyltetrazolium bromide (MTT; 5 mg/ml, 1/10 volume; Sigma‐Aldrich) added to the culture medium at 37°C for 4 h. Then, the culture medium/MTT solution was removed and centrifuged at 10000 rpm for 5 min. After centrifugation, cell pellets were suspended in DMSO. Concomitantly, 300 μl of DMSO was added to each well and thoroughly mixed for 5 min. The optical density values of stained cells (cell pellet and corresponding well) were measured using a microplate reader at 570 nm.

### 2.9 Wound healing assay by live cell imaging

Before each experiment, MDA‐MB‐231 cells were grown to confluence in 96‐well plates in a standard CO_2_ incubator. The 96‐pin IncuCyte WoundMaker was used to simultaneously create precise and reproducible wounds by gently removing cells from the confluent monolayer. After washing, serum‐free HMF CM (SPARC‐immunodepleted or not) was added, plates were placed in the IncuCyte device and cell monolayers were scanned every hour. Wound width, wound confluence and relative wound density were calculated using user‐informed algorithms that are part of the IncuCyte software package. These algorithms identify the wound region and provide visual representations of the segmentation parameters.

### 2.10 Tumor spheroids

To generate tumor spheroids, 5 × 10^3^ MDA‐MB‐231 cells/well were seeded in 150 μl complete medium in ultra‐low attachment 96‐well plates (Corning 96‐well Clear Round Bottom Ultra‐Low Attachment Microplate, NY). Plates were centrifuged at 1000 rpm for 10 min, and 3 days later each spheroid was embedded in collagen gel that included 1× DMEM, penicillin and streptomycin, 2% of SPARC‐immunodepleted FCS, 3.75 g/L sodium bicarbonate, 20 mM Hepes, 1 mg/ml rat collagen I and 1.5 mM NaOH (qsp 150 μl/well in H_2_O). After 30 min at 37°C, serum‐free HMF CM (SPARC‐immunodepleted or not) was added on the spheroid‐containing polymerized collagen gel. MDA‐MB‐231 cell invasion area was analyzed in representative images with ImageJ.

### 2.11 Statistical analyses

Continuous variables were reported using medians and range and compared using the Kruskal‐Wallis test. Categorical variables were reported as numbers of observations and frequencies, and compared using the Pearson's chi‐square test or Fisher's exact test (if appropriate). All tests were two‐sided and *P* values <.05 were considered significant. Relapse‐free survival (RFS) and OS were estimated using the Kaplan‐Meier method and compared with the Log‐rank test. RFS was defined as the time between the date of the first histology analysis and the date of the first recurrence at any site. OS was defined as the time between the date of the first histology analysis and the date of death from any cause. Multivariate analyses were performed using Cox proportional hazard models (the *P* value of the likelihood ratio test is reported). Hazard ratios (HR) are given with their 95% confidence interval (CI). All statistical analyses were performed with the STATA 16.0 software (StatCorp, College Station, TX).

## 3 RESULTS

### 3.1 In TNBC, SPARC is expressed in stromal and tumor cells

**FIGURE 1:**
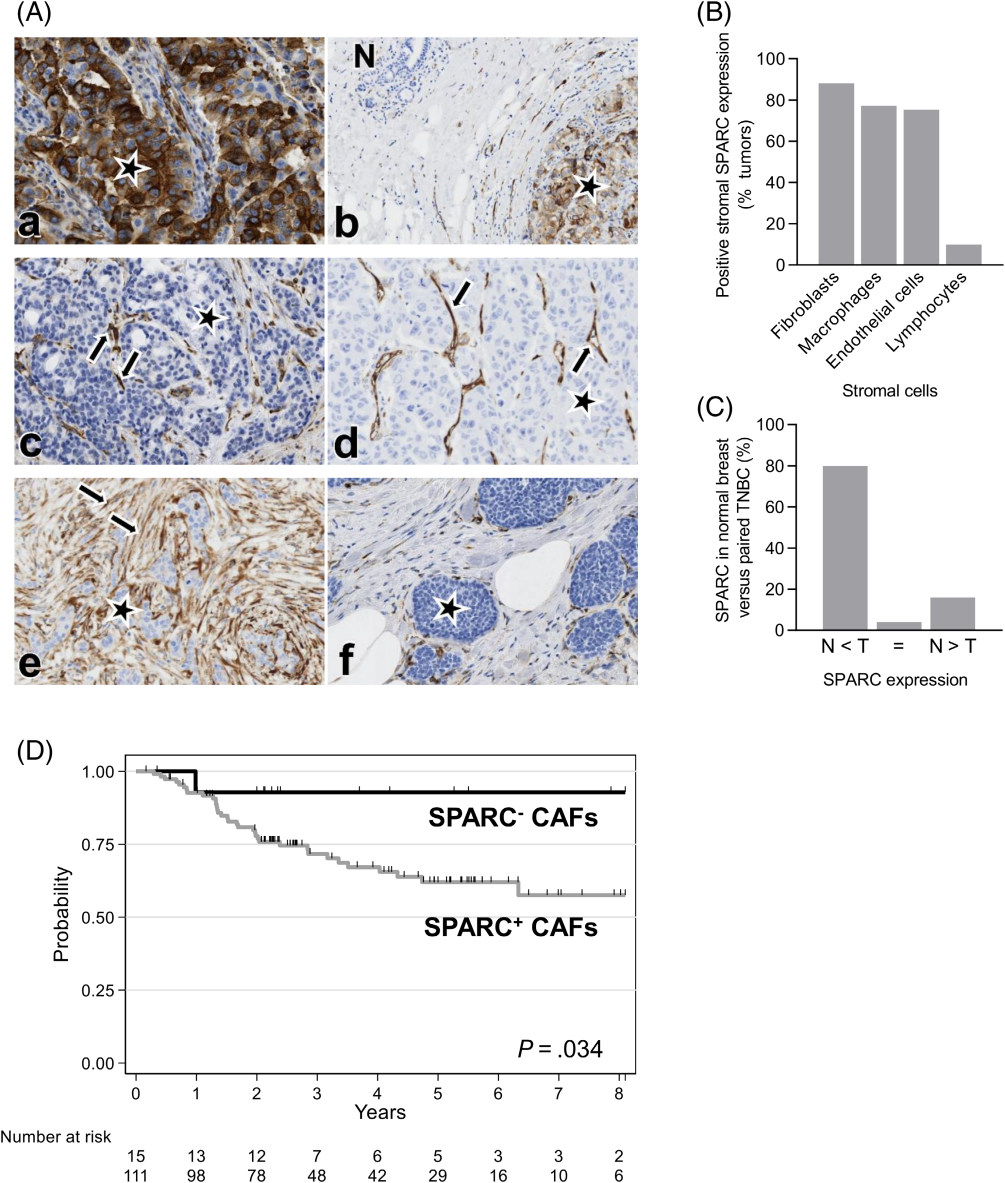
SPARC is a biomarker in TNBC and its expression in CAFs predicts RFS in TNBC. (A) Representative images of TNBC tissue sections showing SPARC expression in cancer cells, CAFs, TAMs, endothelial cells, and in normal breast. SPARC expression was analyzed in a TNBC TMA (n = 148 samples) by IHC using an anti‐SPARC antibody (clone AON‐5031). (a) SPARC expression in tumor cells. (b) Absence of SPARC expression in the adjacent normal breast tissue (N). (c) SPARC expression in TAMs. (d) SPARC expression in endothelial cells. (e) SPARC expression in CAFs. (f) Absence of SPARC expression in CAFs. SPARC scoring in cancer cells: positive (>1% of stained cells), negative (<1% of stained cells). SPARC scoring in stromal cells: positive (>50% of stained cells), negative (<50% of stained cells). Magnification ×200. Stars: tumor cells; arrows: SPARC staining. (B) Quantification of SPARC expression in TNBC stroma. Percentage of TNBC samples with positive SPARC signal (>50% of stained cells) in the indicated stromal cell types. N = 148 samples. (C) Quantification of SPARC expression in normal breast. Percentage of normal breast tissue samples in which SPARC expression was lower (N < T), similar (=) or higher (N > T) than in the adjacent TNBC. T, tumor; N, normal breast; n = 50 samples. (D) Relapse‐free survival according to SPARC expression status in CAFs. Patients with TNBC were divided in two subgroups according to SPARC expression in CAFs: SPARC^+^ CAFs and SPARC^−^ CAFs

To determine SPARC expression in TNBC (tumor and stroma), TMAs were generated using samples from 148 patients with TNBC (Table 1). Their median age was 61.5 years (range 30.2‐98.6), and 68.2% of them received adjuvant chemotherapy. Most TNBC (52.7%) were pT2, and 60.8% pN0. Moreover, 85.5% of tumors were ductal carcinomas, 6.9% lobular carcinomas, and 7.6% other histological types; 11% of tumors were classified as Scarff‐Bloom‐Richardson histological grade 1‐2. A basal‐like phenotype was observed in 61.9% of samples, and 66.9% of tumors expressed PD‐L1. In 51.7% of tumors, TAMs were more abundant than TILs, and > 20% of fibrosis was observed in 74.4% of tumors. SPARC expression (>50% of stained cells) in CAFs, TAMs, endothelial cells and TILs was detected in 88.1%, 77.1%, 75.2% and 9.8% of TNBC samples, respectively (Figure 1A, B and Table 1). SPARC staining in tumor cells (>1% stained tumor cells) was observed in 42.4% of TNBC samples (Figure 1A, Table 1). In 80% of samples, SPARC expression was lower in the adjacent normal breast tissue than in the tumor tissue (Figure 1A, C).

**TABLE 1:** Clinicopathological characteristics of the whole TNBC population and SPARC expression status in cancer and stromal cells

Clinical and tumor characteristics	Whole population (N = 148)	Clinical and tumor characteristics	Whole population (N = 148)
		SPARC expression in TAMs	
Age (years), median [min‐max]	61.5 [30.2‐98.6]	Negative	27 (22.9%)
<55 years	51 (34.5%)	Positive	91 (77.1%)
≥55 years	97 (65.5%)	Missing	30
Tumor size		SPARC expression in endothelial cells	
T1	52 (35.1%)	Negative	27 (24.8%)
T2	78 (52.7%)	Positive	82 (75.2%)
T3/T4	18 (12.2%)	Missing	39
Nodal status		SPARC expression in TILs	
N−	90 (60.8%)	Negative	74 (90.2%)
N+	58 (39.2%)	Positive	8 (9.8%)
		Missing	66
Histological grade (SBR)		TIL density	
1‐2	16 (11.0%)	[0‐1]	42 (29.6%)
3	130 (89.0%)	>1	100 (70.4%)
Missing	2	Missing	6
Histology		PD‐L1 expression in tumor cells	
Ductal	124 (85.5%)	<1%	45 (33.1%)
Lobular	10 (6.9%)	≥1%	91 (66.9%)
Other	11 (7.6%)	Missing	12
Missing	3		
Adjuvant chemotherapy		PD‐L1 expression in TILs	
No	47 (31.8%)	0	20 (14.9%)
Yes	101 (68.2%)	[0‐10]	32 (23.9%)
		[10‐50]	40 (29.9%)
		≥ 50	42 (31.3%)
		Missing	14
Basal‐like phenotype		PD1 expression in TILs	
≤10%	56 (38.1%)	0	18 (12.9%)
Basal	91 (61.9%)	<10	30 (21.3%)
Missing	1	[10‐50]	74 (52.9%)
		≥50	18 (12.9%)
		Missing	8
SPARC expression in tumor cells		Fibrosis	
Negative	76 (57.6%)	0	4 (3.0%)
Positive	56 (42.4%)	< 20%	31 (22.6%)
Missing	16	20%‐50%	27 (19.7%)
		>50%	75 (54.7%)
		Missing	11
SPARC expression in CAFs		TAMs (inflammation)	
Negative	15 (11.9%)	0/1	25 (17.5%)
Positive	111 (88.1%)	2	44 (30.8%)
Missing	22	3	74 (51.7%)
		Missing	5

Abbreviations: CAFs, cancer‐associated fibroblasts; SBR, Scarff‐Bloom‐Richardson; TAMs, tumor‐associated macrophages; TILs, tumor‐infiltrating lymphocytes.

### 3.2 SPARC expression in CAFs predicts RFS in patients with TNBC

As SPARC was expressed in the tumor and stromal compartments, its prognostic value was then evaluated. The median follow‐up time was 5.4 years (range [0.1‐14.3]). Local or regional recurrence occurred in 10 (7%) patients, and metastases (alone or with loco‐regional recurrence) in 32 (22.5%) patients. RFS was not different in patients with SPARC‐positive (SPARC^+^) and SPARC‐negative (SPARC^−^) tumor cells (Table 2 and Figure S1). Conversely, RFS was lower in patients with SPARC^+^ than SPARC^−^ CAFs (HR = 5.09, 95% CI [0.70‐37.18], *P* = .034) (Table 2 and Figure 1D). Moreover, RFS tended to be better in patients with SPARC^+^ than SPARC^−^ TAMs (HR = 0.52, 95% CI [0.25‐1.07], *P* = .088) (Table 2 and Figure S2). SPARC expression status in endothelial cells (Figure S3) and TILs (Figure S4) did not have any prognostic value (Table 2). In univariate analysis, tumor size, nodal status, adjuvant chemotherapy and SPARC expression in CAFs were correlated with RFS (Table 2). In multivariate analysis, only nodal status (HR = 2.96, 95% CI [1.48‐5.94], *P* = .001), adjuvant chemotherapy (HR = 0.35, 95% CI [0.18‐0.68], *P* = .002) and SPARC expression in CAFs (HR = 6.17, 95% CI [0.84‐45.2], *P* = .015) were independent prognostic factors of RFS (Table 2). During the follow‐up, 46 (31.1%) patients died among whom 11 (7.4%) without any TNBC recurrence. In univariate analysis, age (*P* = .027), tumor size (*P* < .001), nodal status (*P* = .002) and adjuvant chemotherapy (*P* = .006) were associated with OS (Table S1). In multivariate analysis, only tumor size (*P* = .05), nodal status (*P* = .008) and adjuvant chemotherapy (*P* < .001) were independent prognostic factors of OS (Table S1). Patients with SPARC^+^ CAFs (n = 111, 88.1%) were younger (38.7% vs 6.7%; *P* = .018) and tended to have ductal tumors (88.0% vs 73.3%; *P* = .08) compared with patients with SPARC^−^ CAFs (Table S2). In addition, SPARC^+^ TAMs and SPARC^+^ endothelial cells were detected more frequently in patients with SPARC^+^ than SPARC^−^ CAFs (80.6% vs 41.7%, *P* = .007, and 78.0% vs 50%, *P* = .026, respectively) (Table S2). Fibrosis (>50%) was significantly less frequent in patients with SPARC^+^ than SPARC^−^ CAFs (48.6% vs 80%; *P* = .028) (Table S2). PD‐L1 expression (>50%) in TILs was more frequently detected in patients with SPARC^+^ than SPARC^−^ CAFs (34.8% vs 15.4%; *P* = .049) (Table S2). TIL density, PD‐L1 expression in tumor cells and PD‐1 expression in TILs were not significantly different between patients with SPARC^+^ and SPARC^−^ CAFs (Table S2).

**TABLE 2:** Univariate and multivariate Cox proportional hazard models to identify prognostic factors of recurrence‐free survival (RFS) in TNBC

Clinical and tumor characteristics	Univariate analysis	Multivariate analysis
	HR 95% CI	HR 95% CI
	N = 148	N = 126
Age	N = 148	
<55 years	1	
≥55 years	1.52 [0.77‐3.03]	
	*P* = .214	
Tumor size	N = 148	
T1	1	
T2	1.67 [0.74‐3.75]	
T3/T4	5.08 [2.07‐12.47]	
	***P* = .002**	
Nodal status	N = 148	
N−	1	1
N+	2.77 [1.49‐5.14]	2.96 [1.48‐5.94]
	***P* = .001**	***P* = .001**
Histological grade (SBR)	N = 146	
1‐2	1	
3	0.82 [0.36‐1.85]	
	*P* = .645	
Histology	N = 145	
Ductal	1	
Lobular	1.51 [0.59‐3.86]	
Other	0.77 [0.19‐3.21]	
	*P* = .651	
Adjuvant chemotherapy	N = 148	
No	1	1
Yes	0.43 [0.24‐0.78]	0.35 [0.18‐0.68]
	***P* = .007**	***P* = .002**
Basal‐like phenotype	N = 147	
Yes	1	
No	1.55 [0.85‐2.83]	
	*P* = .152	
SPARC expression in tumor cells	N = 132	
Negative	1	
Positive	0.84 [0.44‐1.62]	
	*P* = .599	
SPARC expression in CAFs	N = 126	
Negative	1	1
Positive	5.09 [0.70‐37.18]	6.17 [0.84‐45.2]
	***P* = .034**	***P* = .015**
SPARC expression in TAMs	N = 118	
Negative	1	
Positive	0.52 [0.25–1.07]	
	*P* = .088	
SPARC expression in endothelial cells	N = 109	
Negative	1	
Positive	0.59 [0.29‐1.21]	
	*P* = .165	
SPARC expression in TILs	N = 82	
Negative	1	
Positive	0.81 [0.19‐3.46]	
	*P* = .769	
TIL density	N = 142	
[0‐1]	1	
>1	0.92 [0.48‐1.77]	
	*P* = .807	
PD‐L1 expression in tumor cells	N = 136	
<1%	1	
≥1%	0.74 [0.39‐1.40]	
	*P* = .360	
PD‐L1 expression in TILs	N = 134	
0	1	
[0‐50]	2.20 [0.66‐7.40]	
≥50	2.12 [0.60‐7.52]	
	*P* = .356	
PD1 expression in TILs	N = 140	
0	1	
[0‐50]	1.28 [0.46‐3.64]	
≥50	0.80 [0.20‐3.21]	
	*P* = .593	
Fibrosis	N = 137	
≤50%	1	
>50%	0.98 [0.52‐1.83]	
	*P* = .948	
TAMs (inflammation)	N = 143	
0/1	1	
2	1.97 [0.78‐4.96]	
3	1.14 [0.46‐2.86]	
	*P* = .180	

*Note*: *p* value in bold, statistically significant.

Abbreviations: CAFs, cancer‐associated fibroblasts; CI, confidence interval; HR, hazard ratio; SBR, Scarff‐Bloom‐Richardson; TAMs, tumor‐associated macrophages; TILs, tumor‐infiltrating lymphocytes.

### 3.3 SPARC expression in TNBC cytosols, PDX and cell lines

**FIGURE 2:**
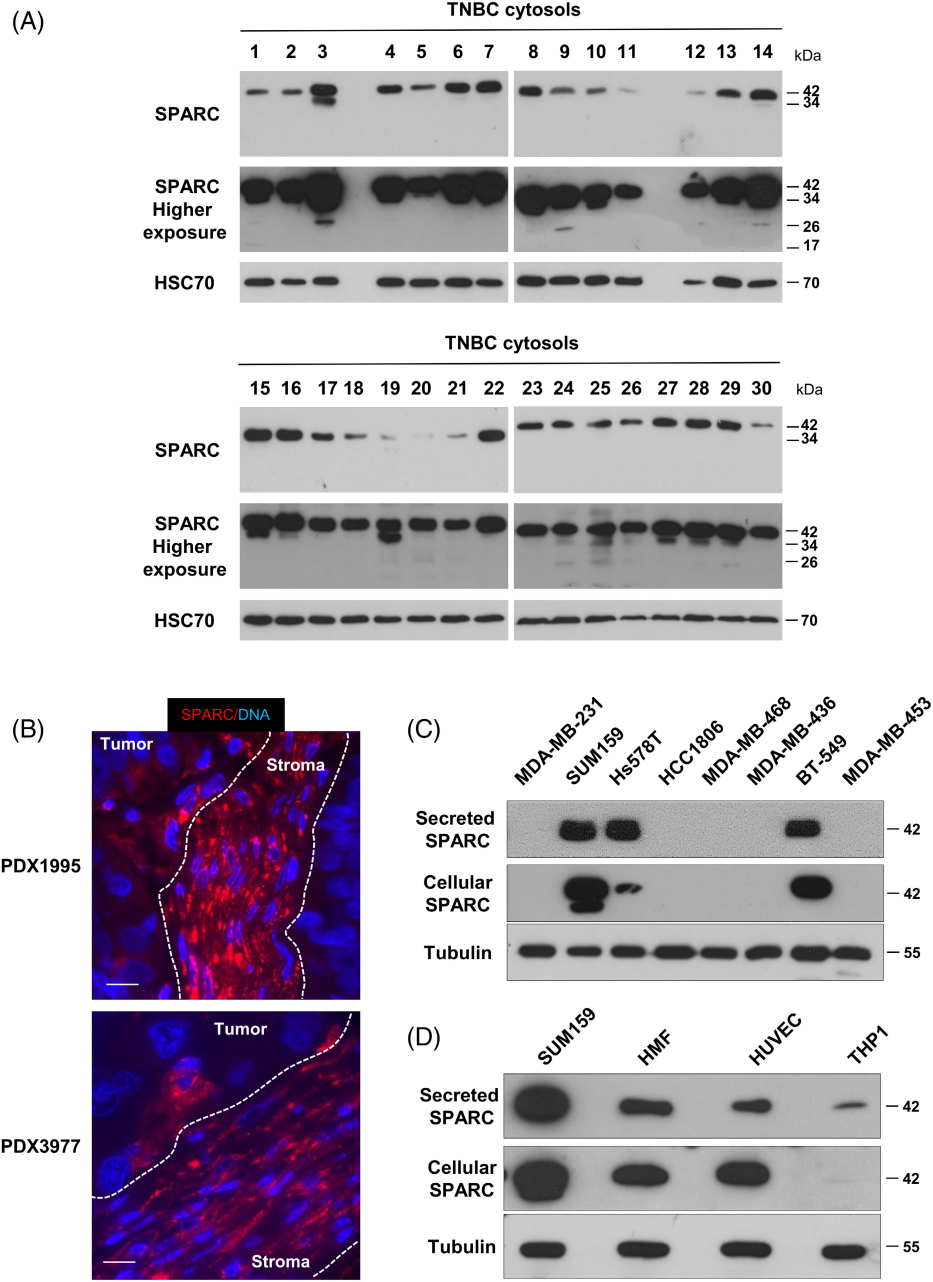
SPARC expression in TNBC cytosols, PDX, and cell lines. (A) SPARC expression in TNBC cytosols. SPARC expression was determined in 30 cytosols from primary TNBC samples. Whole cytosols (20 μg proteins) were analyzed by 13.5% SDS‐PAGE and immunoblotting with an anti‐SPARC antibody (clone AON‐5031). A higher exposure of SPARC is shown. HSC70 (clone B‐6) was used as loading control. (B) SPARC expression and localization in TNBC PDX. PDX B1995 and PDX B3977 sections were incubated with an anti‐SPARC polyclonal antibody (15274‐1‐AP) (red). Nuclei were stained with Hoechst 33342 (blue). Scale bar, 10 μm. (C) SPARC expression and secretion in TNBC cell lines. Whole cell extracts (30 μg proteins) and serum‐free 24 h conditioned media (40 μl) from the indicated TNBC cell lines were separated on 13.5% SDS‐PAGE and analyzed by immunoblotting with an anti‐SPARC (clone AON‐5031) antibody. Tubulin was used as loading control. (D) SPARC expression and secretion in stromal cell lines. Whole cell extracts (30 μg proteins) and serum‐free 24 h conditioned media (40 μl) from the indicated cell lines were separated on 13.5% SDS‐PAGE and analyzed by immunoblotting with an anti‐SPARC (clone AON‐5031) antibody. Tubulin was used as loading control

To further validate SPARC expression in TNBC, its expression was assessed in the cytosols of 30 primary TNBC samples by western blot analysis. SPARC protein was detected in all cytosols and SPARC cleaved fragments in about 30% of samples (Figure 2A). SPARC protein expression and localization were then examined in two TNBC PDXs (PDX B1995 and PDX B3977).^41^ SPARC was localized in stromal cells, including CAFs, in the extracellular matrix and in some tumor cells (Figure 2B). Next, SPARC expression and secretion were analyzed in TNBC and stromal cell lines. SPARC was expressed and secreted by three of the eight TNBC cell lines tested (SUM159, Hs578T, BT‐549) that exhibit a basal‐like phenotype (Figure 2C). SPARC was also expressed and secreted by HMFs, and to a lesser extent by HUVECs and M2‐polarized THP1 macrophages (Figure 2D and Figure S5).

### 3.4 SPARC is expressed in different CAF subsets

**FIGURE 3:**
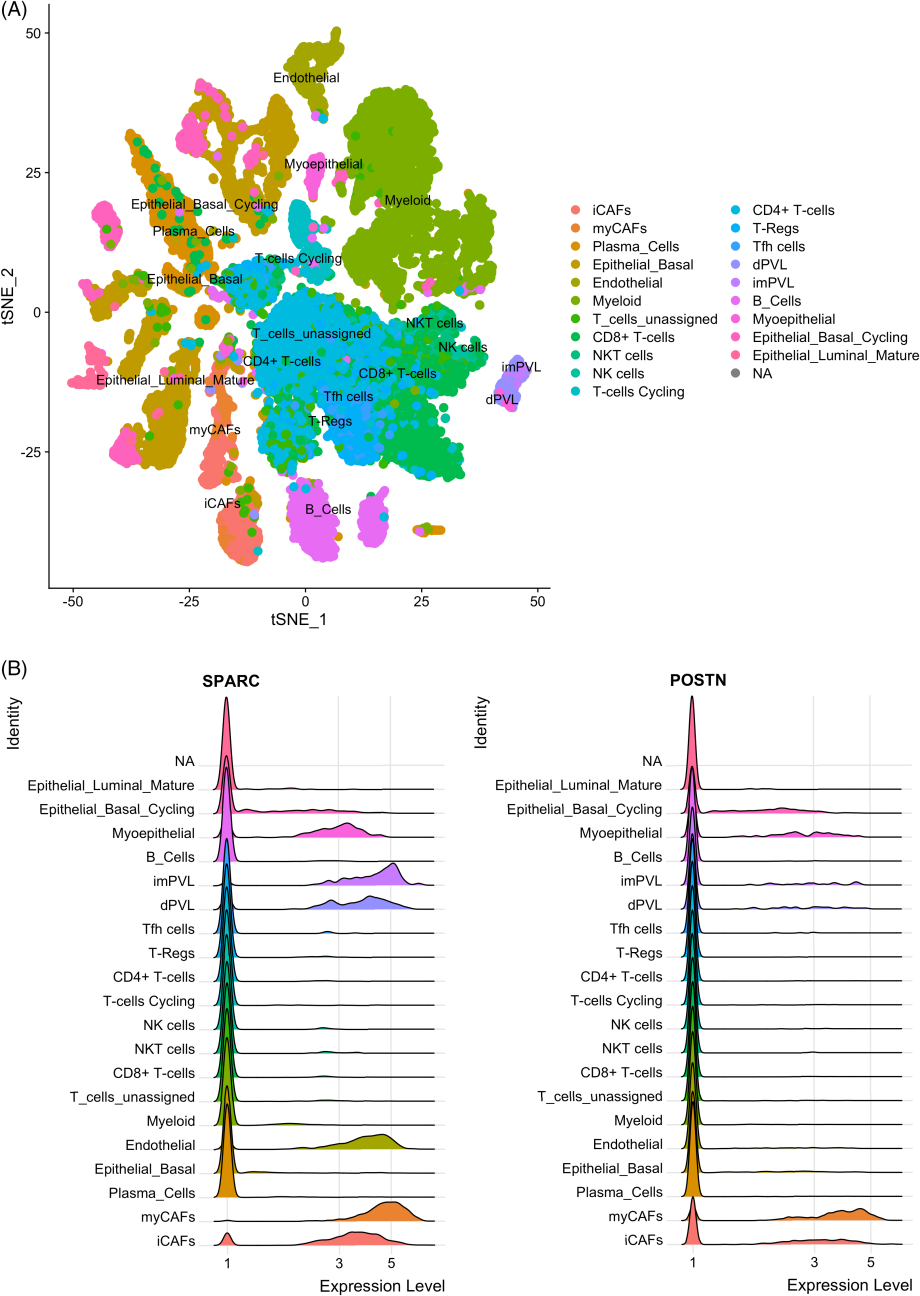
Expression of *SPARC* and *POSTN* mRNAs in TNBC by single‐cell RNA‐seq data analysis. (A) Cell populations. Twenty cell populations were identified by analysis of the previously published single‐cell RNA‐seq dataset PRJEB35405 that included five patients with TNBC, according to.^9^ (B) *SPARC* and *POSTN* mRNA expression. Relative expression of *SPARC* and *POSTN* mRNA in each of the 20 populations identified by single‐cell RNA‐seq analysis, according to.^9^ MyCAFs, myofibroblast‐like CAFs; iCAFs, inflammatory‐like CAFs; endothelial, endothelial cells; dPVL, differentiated perivascular‐like cells; imPVL, immature perivascular‐like cells; myoepithelial, myoepithelial cells; epithelial basal cycling, cancer cells

Based on the finding that SPARC expression in CAFs predicts RFS in TNBC, SPARC expression in different CAF subpopulations was thoroughly investigated through meta‐analysis of recently published scRNA‐seq data from patients with TNBC.^9^, ^10^, ^11^ In the first dataset (n = 5 patients with TNBC),^9^ the t‐distributed Stochastic neighbor embedding (tSNE) technique identified 20 different cell populations, including two fibroblastic cell populations, the first with features of myofibroblasts (myCAFs), and the second with an inflammatory phenotype (iCAFs) characterized by high expression of growth factors and immunomodulatory molecules (Figure 3A). The scRNA‐seq data analysis^9^ showed that *SPARC* mRNA was strongly expressed in myCAFs and iCAFs, as well as *POSTN* (the gene encoding periostin, a CAF‐secreted protein that promotes cancer progression and chemoresistance)^47^ (Figure 3B). *SPARC* was also detected in perivascular endothelial cells, myoepithelial cells and basal cancer cells^9^ (Figure 3B, Figure S6), in accordance with our TMA analysis (Table 1). In the second scRNA‐seq dataset (n = 6 patients with TNBC),^10^ high *SPARC* and *POSTN* mRNA levels were detected in three distinct CAF subtypes, in endothelial cells, M2‐polarized macrophages and cancer cells (where expression varied in function of the patient) (Figure S7), consistent with our TMA data (Table 1). As these two meta‐analysis indicated that *SPARC* was expressed in different CAF subtypes, another scRNA‐seq dataset (n = 8 patients with breast cancer) that identified different myCAF and iCAF clusters was analyzed.^11^ *SPARC* and *POSTN* mRNAs were detected mainly in myCAFs (ECM‐myCAF, TGFβ‐myCAF, Wound‐myCAF, IFNαβ‐myCAF, Acto‐myCAF clusters) and also in iCAFs (IFNγ‐iCAF, IL‐iCAF, detox‐iCAF clusters) (Figure S8). Altogether, this meta‐analysis highlighted that *SPARC* mRNA is expressed by different CAF subtypes, including myofibroblasts and inflammatory‐like CAFs involved in different tumor‐related processes, such as matrix remodeling, inflammation and resistance to therapy in TNBC.^9^, ^11^ To complement the scRNA‐seq findings, the localization of SPARC and periostin was investigated in the TNBC PDX B1995 microenvironment. Co‐labeling with anti‐SPARC and anti‐periostin antibodies showed that SPARC (in green) partially co‐localized with periostin (in red) in CAFs at the cancer cell‐stromal interface (Figure S9).

### 3.5 Fibroblast‐secreted SPARC affects TNBC cell adhesion, migration and invasion

**FIGURE 4:**
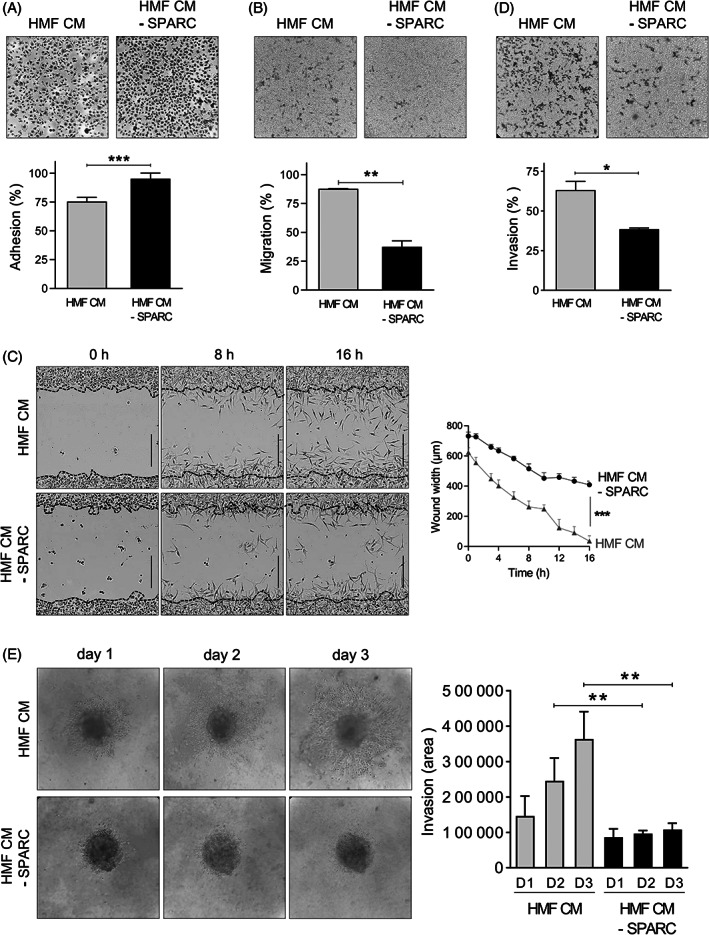
Effects of fibroblast‐secreted SPARC on TNBC cell adhesion, migration and invasion. (A) Cell adhesion. MDA‐MB‐231 cells were let to adhere on a fibronectin matrix in the presence of HMF conditioned medium (HMF CM) or SPARC‐immunodepleted HMF CM (HMF CM—SPARC) for 30 min. Upper panels, representative images of adherent cells stained with crystal violet. Lower panel, adhesion was quantified at 570 nm. Data are the mean (% of seeded cells) ± SD (n = 5); ***P < .001 (Student's *t* test). Similar results were obtained in three independent experiments. (B) Cell migration. MDA‐MB‐231 cells were let to migrate for 16 h on a fibronectin matrix in the presence of HMF CM or SPARC‐immunodepleted HMF CM (HMF CM ‐ SPARC). Upper panels, representative images of migrating cells stained with crystal violet. Lower panels, quantification of migrating MTT‐stained cells (absorbance was read at 570 nm). Data are the mean (% of seeded cells) ± SD (n = 3); **P < .01 (Student's *t* test). Similar results were obtained in three independent experiments. (C) Cell migration induced by wound healing. MDA‐MB‐231 sub‐confluent cell layers were wounded using the 96‐well IncuCyte scratch wound assay. Left panels, representative images of MDA‐MB‐231 wound healing over time (*t* = 0 h, *t* = 6 h, *t* = 16 h) in the presence of HMF CM or SPARC‐immunodepleted HMF CM (HMF CM ‐ SPARC). In the left panels, the initial scratch wound is delimited by the dashed lines. Bars, 400 μm. Right panel, wound healing (wound width, in μm) in the presence of HMF CM or SPARC‐immunodepleted HMF CM (HMF CM ‐ SPARC) was quantified over time. The data are the mean ± SD (n = 3); ***P < .001 (Student's *t* test). Similar results were obtained in another independent experiment. (D) Cell invasion. MDA‐MB‐231 cells were let to invade on a Matrigel matrix in the presence of HMF CM or SPARC‐immunodepleted HMF CM (HMF CM ‐ SPARC) for 16 h. Upper panels, representative images of invading cells stained with crystal violet. Lower panels, invading cells were stained with MTT and quantified at 570 nm. Data are the mean (% of seeded cells) ± SD (n = 3); ***P < .001 (Student's *t* test). Similar results were obtained in three independent experiments. (E) Cell invasion in tumor spheroid assay. MDA‐MB‐231 tumor spheroids embedded in collagen I gel were let to invade in the presence of HMF CM or SPARC‐immunodepleted HMF CM (HMF CM ‐ SPARC) for 3 days. Left panels, representative images of invading MDA‐MB‐231 cells. Right panel, the invading MDA‐MB‐231 cell area was quantified using Image J. Data are the mean ± SD (n = 5); **P < .01 (Student's *t* test).

To obtain some insights into the pathophysiological relevance of SPARC^+^ CAFs in TNBC, the effects on TNBC cell adhesion, motility, wound healing and invasiveness of SPARC‐secreting HMF CM were investigated (Figure S10). The adhesion of MDA‐MB‐231 cells on fibronectin was reduced by 1.3‐fold (*P <* .001) after incubation with HMF CM compared with SPARC‐immunodepleted HMF CM (Figure 4A). Cell motility analysis in Boyden chambers showed that 88% of MDA‐MB‐231 cells passed through the fibronectin‐coated filters after incubation with HMF CM (Figure 4B). Motility was reduced by 2.3‐fold when cells were incubated with SPARC‐immunodepleted CM (Figure 4B; *P* < .01). Moreover, wound healing was significantly faster in MDA‐MB‐231 cells incubated with HMF CM than with SPARC‐immunodepleted CM: wound closure was nearly complete after 16 h in the presence of HMF CM (Figure 4C). Lastly, MDA‐MB‐231 cell invasion through Matrigel‐coated filters in Boyden chambers was 1.6‐fold higher in the presence of HMF CM than SPARC‐immunodepleted CM (Figure 4D; *P* < .05). The capacity of HMF‐secreted SPARC to enhance MDA‐MB‐231 cell invasion was confirmed in a tumor spheroid assay (Figure 4E). MDA‐MB‐231 tumor spheroid invasiveness at day 3 was 3.4‐fold higher in the presence of HMF CM than SPARC‐immunodepleted CM (Figure 4E; *P <* .01). Thus, HMF‐secreted SPARC inhibits adhesion and promotes motility, wound healing and invasion of MDA‐MB‐231 TNBC cells, highlighting its pro‐tumor role.

## 4 DISCUSSION

Here, we showed that in TNBC, SPARC is expressed in both tumor and stromal cells, and that its expression in CAFs independently predicts RFS in patients with TNBC. Previous studies reported that SPARC is overexpressed in TNBC compared with other breast cancer molecular subtypes.^48^, ^49^ In our study using IHC, SPARC expression in tumor cells was detected in 42% of TNBC samples, in agreement with previous literature data (SPARC expression in 37 to 52% of TNBC).^32^, ^48^, ^49^ However, SPARC expression in TNBC has never been correlated with clinicopathological parameters, such as age, histopathologic grade, tumor size and lymph node metastasis.^32^, ^48^ Watkins et al reported that in breast cancer, SPARC is detected more frequently in ductal carcinomas.^30^ Similarly, we found that ductal carcinoma tended to be more frequent in patients with SPARC^+^ CAFs, and that patients with TNBC with SPARC^+^ CAFs were often younger.^50^ SPARC (mRNA or protein) overexpression prognostic value is controversial in TNBC. High SPARC expression in TNBC has been associated with poor prognosis in some studies,^32^, ^34^, ^51^ and with better prognosis in another.^48^ We recently showed that high *SPARC* mRNA expression (n = 225 patients with TNBC) tends to be associated with shorter RFS using an on line survival tool.^40^, ^52^ In our current TNBC population, SPARC expression by tumor cells was not associated with RFS or OS. Studies using IHC reported that SPARC expression in tumor cells was associated with prognosis.^32^, ^53^ Here, we found that SPARC was mainly expressed by stromal cells, including CAFs, and that its expression in CAFs was an independent prognostic factor of poor RFS in TNBC. In patients with SPARC^+^ CAFs, TILs more frequently expressed PD‐L1, suggesting the interest to specifically evaluate the benefit of combining anti‐PD1 or ‐PD‐L1 with anti‐SPARC targeted therapies in this TNBC subgroup. Moreover, fibrosis was less frequent in TNBC samples with SPARC^+^ CAFs, suggesting a better drug accessibility in this TNBC subgroup.^54^ Other studies^50^ reported a frequent SPARC stromal expression, but none, to our knowledge, evaluated its prognostic value or determined SPARC expression in the different stromal cell types.

Here, we observed the presence of SPARC cleaved fragments in about 30% of TNBC cytosols. The anti‐SPARC antibody (clone AON‐5031) used for IHC recognizes full‐length SPARC and also some SPARC N‐terminal fragments. Therefore, the prognostic value of SPARC expression in CAFs in TNBC described in the present study could be explained by the activity of the full‐length protein and also of some of its cleaved fragments. SPARC includes three different structural and functional modules: the N‐terminal acidic domain, the follistatin‐like domain and the C‐terminal extracellular Ca^2+^ binding domain.^22^ SPARC biological activity can be modulated by limited proteolysis, leading to the unmasking of distinct or amplified biological functions compared with those of the full‐length protein.^21^, ^55^ Matrix metalloproteinases (MMP‐1, −2, −3, −9 and − 13) cleave SPARC in vitro in its N‐terminal acid domain and in its extracellular Ca^2+^ binding domain, releasing fragments that have higher affinity for collagens and that modulate cell‐cell and cell‐matrix extracellular interactions in the tumor microenvironment.^56^ Moreover, MMP‐3‐mediated SPARC cleavage in vitro produces fragments that affect angiogenesis.^57^ Cleavage of SPARC extracellular Ca^2+^ binding domain by MMP‐8 and MMP‐13 has been detected in the serum of patients with lung cancer, suggesting their presence also in vivo.^58^ Similarly, cathepsin K cleaves SPARC in vitro and in vivo in its N‐terminal acid domain and in its extracellular Ca^2+^ binding domain in mice harboring prostate cancer bone metastases.^59^ We recently reported that secreted SPARC is cleaved by cathepsin D in TNBC, releasing a 9‐kDa SPARC fragment with enhanced oncogenic properties.^40^

The meta‐analysis of previously published scRNA‐seq datasets^8^, ^9^, ^10^, ^11^ showed that SPARC is expressed by different CAF subsets in TNBC. CAFs are the most abundant stromal cells in many cancers, including TNBC, and they are a phenotypically heterogeneous population, generally described as having a myofibroblastic phenotype (ie, secretory and contractile cells that express α‐SMA). Recently, it was found that fibroblast heterogeneity occurs in breast cancers and in TNBC.^8^, ^9^, ^10^, ^11^ Two myofibroblastic subsets (CAF‐S1 and CAF‐S4) differentially accumulate in TNBC.^8^ CAF‐S1 cells promote an immunosuppressive microenvironment,^8^ whereas CAF‐S4 cells have pro‐metastatic function.^60^ More recently, a scRNA‐seq approach in breast cancer identified eight clusters within the immunosuppressive CAF‐S1 subset, subdivided in myofibroblast‐like and inflammatory‐like CAFs.^11^ Another scRNA‐seq‐based study identified myofibroblast‐like and inflammatory‐like CAFs with immunomodulatory properties in TNBC.^9^ By reanalyzing these scRNA‐seq datasets,^9^, ^10^, ^11^ we noticed that *SPARC* mRNA was expressed by different CAF subsets, especially myofibroblast‐like and inflammatory‐like CAFs, as well as *POSTN*, a gene encoding periostin, a protein that is secreted by CAFs with pro‐tumor activity in breast cancer.^47^ We then confirmed that SPARC and periostin (partially) co‐localize in CAFs within the TNBC PDX microenvironment. Future studies will determine whether SPARC participates in the homeostasis of these different CAF subpopulations in TNBC, and whether SPARC has a different prognostic value when expressed in the different CAF subgroups in TNBC.

In TNBC, CAFs regulate a number of tumor‐promoting processes, including motility and invasion, drug resistance, inflammation and immunosuppression.^8^, ^9^, ^60^, ^61^, ^62^ Our results showed that SPARC secreted by fibroblasts acts directly on TNBC cells by inhibiting their adhesion and promoting/facilitating their motility and invasiveness. It has been reported that SPARC regulates signaling pathways that influence epithelial‐to‐mesenchymal transition, cell adhesion, motility and invasiveness of cancer cells.^23^, ^63^ Moreover, SPARC activation of the ERK and AKT downstream signaling pathways modulates cancer cell adhesion, motility and invasion.^64^ SPARC can bind directly to integrin receptors (αvβ1, αvβ3 and αvβ5), resulting in activation of the intracellular kinase Akt, the focal adhesion kinase FAK and the integrin‐related kinase ILK.^65^, ^66^ Future mechanistic studies should decipher the signaling pathways affected by CAF‐secreted SPARC in TNBC cells. All these findings suggest that SPARC may be a therapeutic target in TNBC. Drugs that target CAFs have emerged as an important option for improving cancer therapies, and targeting CAF‐derived extracellular matrix proteins has been proposed as an innovative anti‐stromal therapy.^67^ Our work strongly suggests that CAF‐derived SPARC also may be a promising candidate for anti‐stromal therapy.

## 5 CONCLUSION

In this series, almost 88.1% of TNBC harbored SPARC^+^ CAFs and displayed distinct clinicopathological characteristics. SPARC expression in CAFs independently predicted worse RFS. This biomarker could be useful to identify a specific TNBC subgroup with worse prognosis. Furthermore, SPARC was expressed by different CAF subpopulations in TNBC, and fibroblast‐secreted SPARC exhibited pro‐tumor functions. Our results could have therapeutic implications for future anti‐SPARC^+^ CAF targeted therapy.

**Abbreviations**
BM40: basement membrane 40
BSA: bovine serum albumin
CAFs: cancer‐associated fibroblasts
Cath‐D: Cathepsin D
CI: confidence interval
CM: conditioned medium
dPVL: differentiated perivascular‐like cells
ECM: extracellular matrix
EGFR: epithelial growth factor receptor
ER: estrogen receptor
FCS: fetal calf serum
HES: hematoxylin‐eosin‐safranin
HMF: human mammary fibroblast
HR: hazard ratios
HUVECs: human umbilical vein endothelial cells
iCAFs: inflammatory‐like CAFs
IFN: interferon
IHC: immunohistochemistry
IL: interleukin
myCAFs: myofibroblasts‐like CAFs
OS: overall survival
PD‐1: programmed cell death 1
PD‐L1: programmed cell death ligand 1
PDX: patient‐derived xenograft
PR: progesterone receptor
PVL: immature perivascular‐like cells
RFS: relapse‐free survival
scRNA‐seq: single‐cell RNA sequencing
SPARC: secreted protein acidic and rich in cysteine
TAMs: tumor‐associated macrophages
TILs: tumor‐infiltrating lymphocytes
TMA: tissue microarray
TNBC: triple negative breast cancer
TNF: tumor necrosis factors
tSNE: t‐distributed stochastic neighbor embedding

## AUTHOR CONTRIBUTIONS

The work reported in the paper has been performed by the authors, unless clearly specified in the text. Lindsay B. Alcaraz, Aude Mallavialle, Andrei Turtoi, Pascal Roger, Séverine Guiu, Emmanuelle Liaudet‐Coopman designed the experiments and prepared the manuscript. Lindsay B. Alcaraz, Aude Mallavialle, Florence Boissière‐Michot, Hanane Mansouri, Joelle Simony‐Lafontaine, Andrei Turtoi, Pascal Roger performed the experiments. Lindsay B. Alcaraz, Aude Mallavialle, Caroline Mollevi, Florence Boissière‐Michot, William Jacot, Joelle Simony‐Lafontaine, Andrei Turtoi, Pascal Roger, Séverine Guiu, Emmanuelle Liaudet‐Coopman provided material and analyzed data. Lindsay B. Alcaraz, Aude Mallavialle, Caroline Mollevi, Florence Boissière‐Michot, Joelle Simony‐Lafontaine, Valérie Laurent‐Matha, Thierry Chardès, Andrei Turtoi, Pascal Roger, Séverine Guiu, Emmanuelle Liaudet‐Coopman analyzed data and proof‐read and finalized the manuscript.

## CONFLICT OF INTEREST

The authors declare no conflict of interest.

## ETHICS STATEMENT

For TNBC cytosols, patient samples were processed according to the French Public Health Code (law n°2004‐800, articles L. 1243‐4 and R. 1243‐61), and the biological resources center has been authorized (authorization number: AC‐2008‐700; Val d'Aurelle, ICM, Montpellier) to deliver human samples for scientific research. TNBC samples were provided by the Biological Resource Center (Biobank number BB‐0033‐00059) after approval by the Montpellier Cancer Institute Institutional Review Board (ID number ICM‐CORT‐2016‐04), following the French Ethics and Legal regulations for the patients' information and consent. All patients were informed before surgery that their surgical specimens may be used for research purposes.

## Supporting information


**APPENDIX S1** Supporting information

## Data Availability

Data sources and handling of publicly available datasets are described in the Materials and Methods. The R script used in the current study to generate Figures 3, S6, S7 and S8 was deposited in a public database: https://github.com/DirtyHarry80/BreastCanceR. Further information is available from the corresponding author upon request.
