# Supplementary material for: SPARC in cancer‐associated fibroblasts is an independent poor prognostic factor in non‐metastatic triple‐negative breast cancer and exhibits pro‐tumor activity
Source: Int J Cancer. 2022 Nov 30;152(6):1243–58. doi: 10.1002/ijc.34345 (PMC10099777; doi:10.1002/ijc.34345)
Supplement: Supplementary file 1 — APPENDIX S1 Supporting information [file IJC-152-1243-s001.pdf]

SPARC in cancer-associated fibroblasts is an independent poor prognostic factor in non-metastatic triple-negative breast cancer and exhibits pro-tumor activity

Lindsay B Alcaraz[1#], Aude Mallavialle[1], Caroline Mollevi[2,3], Florence Boissière-Michot[4], Hanane Mansouri[1,5], Joelle Simony-Lafontaine[4], Valérie Laurent-Matha[1], Thierry Chardès[1], William Jacot[1,4,6], Andrei Turtoi[1], Pascal Roger[1,7*], Séverine Guiu[1,6*], Emmanuelle Liaudet-Coopman[1*#]

- Supplementary Material and Methods

RT-qPCR

- Supplementary Tables

Table S1. Univariate and multivariate Cox proportional hazard models to identify prognostic factors of overall survival (OS) in TNBC.

Table S2. Clinicopathological characteristics in function of SPARC expression (SPARC⁺ and SPARC⁻) in CAFs.

- Supplementary Figures

Figure S1. Relapse-free survival in function of the SPARC expression status in TNBC cancer cells.

Figure S2. Relapse-free survival in function of SPARC status in TAMs. Patients with TNBC were

Figure S3. Relapse-free survival according in function of SPARC expression status in endothelial cells.

Figure S4. Relapse-free survival according to SPARC expression status in TILs.

Figure S5. THP1 monocyte differentiation into M2 macrophages

Figure S6. Differential *SPARC* gene expression in different cell populations within TNBC samples by single-cell RNA-seq data analysis.

Figure S7. Expression of *SPARC* and *POSTN* mRNAs in TNBC by single-cell RNA-seq analysis

Figure S8. Expression of *SPARC* and *POSTN* mRNAs in CAF-S1 clusters in breast cancer by single-cell RNA-seq analysis

Figure S9. Co-localization of SPARC with periostin in TNBC PDX

Figure S10. Immunodepletion of SPARC secreted in the conditioned medium from HMFs.

Supplementary Material and Methods

RT-qPCR

RNA was isolated using the RNeasy® Plus Mini Kit (Qiagen, Hilden, Germany) and 1 µg of total RNA was reverse transcribed using the SuperScript™ III reverse transcriptase kit (Invitrogen). Real-time PCR was performed using SYBR® Premix Ex Taq™ (Tli RNaseH Plus) (Takara Clontech) on a Light Cycler 480 SYBR Green I master and a Light Cycler 480 apparatus (both from Roche Diagnostics, Indianapolis, IN) with the following primers: human CD206 forward: 5'-GGGTTGCTATCACTCTCTATG-3'; human CD206 reverse: 5'-TTTCTTGTCTGTTGCCGTAGTT-3'; human GAPDH forward: 5'-GAAGGTCGGAGTCAACGGATT-3'; human GAPDH reverse: 5'-TGACGGTGCCATGGAATTTG-3'. *CD206* expression was normalized to *GAPDH* expression.

Clinical and tumor characteristics	Univariate analysis<br>HR 95% CI<br>N = 148	Multivariate analysis<br>HR 95% CI<br>N = 148
**Age**	N=148	
< 55 years	1	
≥ 55 years	2.21 [1.03-4.75]	
	***P* = 0.027**	
**Tumor size**	N=148	
T1	1	1
T2	3.04 [1.17-7.93]	1.99 [0.74-5.30]
T3/T4	6.73 [2.34-19.38]	3.66 [1.22-11.0]
	***P* < 0.001**	***P* = 0.050**
**Nodal status**	N=148	
N-	1	1
N+	2.47 [1.36-4.47]	2.38 [1.25-4.55]
	***P* = 0.002**	***P* = 0.008**
**Histological grade (SBR)**	N=146	
1-2	1	
3	1.07 [0.45-2.53]	
	P = 0.883	
**Histology**	N=145	
Ductal	1	
Lobular	0.70 [0.22-2.29]	
Other	1.17 [0.42-3.29]	
	P = 0.777	
**Adjuvant chemotherapy**	N=148	
No	1	1
Yes	0.36 [0.20-0.64]	0.33 [0.18-0.60]
	***P* = 0.006**	***P* < 0.001**

**Basal-like phenotype**	N=147	
Yes	1	
No	1.16 [0.64-2.08]	
	P = 0.624	
**SPARC expression in tumor cells**	N=132	
Negative	1	
Positive	0.75 [0.40-1.41]	
	P = 0.363	
**SPARC expression in CAFs**	N=126	
Negative	1	
Positive	1.92 [0.59-6.23]	
	P = 0.235	
**SPARC expression in TAMs**	N=118	
Negative	1	
Positive	0.59 [0.29-1.16]	
	***P* = 0.139**	
**SPARC expression in endothelial cells**	N=109	
Negative	1	
Positive	0.73 [0.35-1.51]	
	P = 0.401	
**SPARC expression in TILs**	N=82	
Negative	1	
Positive	1.10 [0.32-3.75]	
	P = 0.882	
**TIL density**	N=142	
[0-1]	1	
>1	0.94 [0.50-1.75]	
	P = 0.839	
**PD-L1 expression in tumor cells**	N=136	
< 1%	1	
≥ 1%	0.91 [0.49-1.70]	
	P = 0.770	

**PD-L1 expression in TILs**	N=134	
0	1	
]0-50[	1.90 [0.66-5.45]	
≥ 50	1.10 [0.33-3.66]	
	P = 0.238	
**PD1 expression in TILs**	N=140	
0	1	
]0-50[	0.94 [0.40-2.25]	
≥ 50	0.73 [0.22-2.38]	
	P = 0.832	
**Fibrosis**	N=137	
≤ 50%	1	
> 50%	1.10 [0.60-2.02]	
	P = 0.746	
**TAMs (inflammation)**	N=143	
0/1	1	
2	1.11[0.51-2.43]	
3	0.67 [0.31-1.45]	
	P = 0.285	

Table S1. Univariate and multivariate Cox proportional hazard models to identify prognostic factors of overall survival (OS) in TNBC

SBR: Scarff-Bloom-Richardson; CAFs: cancer-associated fibroblasts; TAMs: tumor-associated macrophages; TILs: tumor-infiltrating lymphocytes
HR = hazard ratio; CI = confidence interval; p values in bold, statistically significant.

Clinical and tumor characteristics	SPARC expression in CAFs Negative (N=15)		SPARC expression in CAFs Positive (N=111)		*P value*
**Age**					0.018
< 55 years	1	(6.7%)	43	(38.7%)	
≥ 55 years	14	(93.3%)	68	(61.3%)	
**Tumor size**					0.334
T1	6	(40.0%)	36	(32.4%)	
T2	9	(60.0%)	59	(53.2%)	
T3/T4	0		16	(14.4%)	
**Nodal status**					0.863
N-	9	(60.0%)	64	(57.7%)	
N+	6	(40.0%)	47	(42.3%)	
**Histological grade (SBR)**					0.130
1-2	3	(20.0%)	8	(7.3%)	
3	12	(80.0%)	101	(92.7%)	
**Histology**					0.080
Ductal	11	(73.3%)	95	(88.0%)	
Lobular	3	(20.0%)	5	(4.6%)	
Other	1	(6.7%)	8	(7.4%)	
**Adjuvant chemotherapy**					0.186
No	7	(46.7%)	33	(29.7%)	

Yes	8 (53.3%)	78 (70.3%)	
**Basal-like phenotype**			0.678
≤ 10%	9 (60.0%)	72 (65.4%)	
Basal	6 (40.0%)	38 (34.6%)	
**SPARC expression in tumor cells**			0.603
Negative	8 (53.3%)	67 (60.4%)	
Positive	7 (46.7%)	44 (39.6%)	
**SPARC expression in TAMs**			**0.007**
Negative	7 (58.3%)	20 (19.4%)	
Positive	5 (41.7%)	83 (80.6%)	
**SPARC expression in endothelial cells**			**0.026**
Negative	7 (50.0%)	20 (22.0%)	
Positive	7 (50.0%)	71 (78.0%)	
**SPARC expression in TILs**			1.000
Negative	8 (100.0%)	65 (89.0%)	
Positive	0	8 (11.0%)	
**TIL density**			0.127
[0-1]	6 (42.9%)	26 (23.9%)	
> 1	8 (57.1%)	83 (76.1%)	
**PD-L1 expression in tumor cells**			0.109
< 1%	7 (53.9%)	31 (28.4%)	
≥ 1%	6 (46.1%)	78 (71.6%)	

**PD-L1 expression in TILs**			**0.049**
0	4 (30.8%)	10 (9.2%)	
]0-50[	7 (53.8%)	61 (56.0%)	
≥ 50	2 (15.4%)	38 (34.8%)	
**PD1 expression in TILs**			0.415
0	2 (15.4%)	11 (10.2%)	
]0-50[	8 (61.5%)	83 (76.9%)	
≥ 50	3 (23.1%)	14 (12.9%)	
**Fibrosis**			**0.028**
≤ 50%	3 (20.0%)	56 (51.4%)	
> 50%	12 (80.0%)	53 (48.6%)	
**TAMs (inflammation)**			0.349
0/1	4 (28.6%)	15 (13.8%)	
2	3 (21.4%)	32 (29.4%)	
3	7 (50.0%)	62 (56.9%)	

Table S2. **Clinicopathological characteristics in function of SPARC expression ($SPARC^+$ and $SPARC^-$) in CAFs**

SBR: Scarff-Bloom-Richardson; CAFs: cancer-associated fibroblasts; TAMs: tumor-associated macrophages; TILs: tumor-infiltrating lymphocytes.

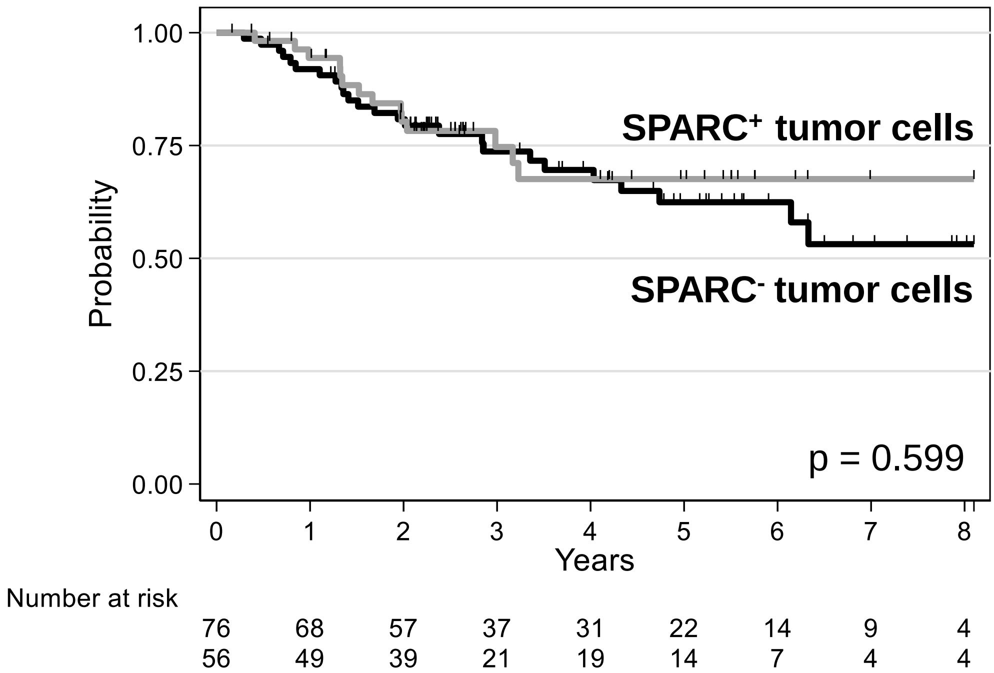

Figure S1. Relapse-free survival in function of the SPARC expression status in TNBC cancer cells.

Patients with TNBC were divided in two subgroups according to SPARC expression in tumor cells: $SPARC^{+}$ and $SPARC^{-}$.

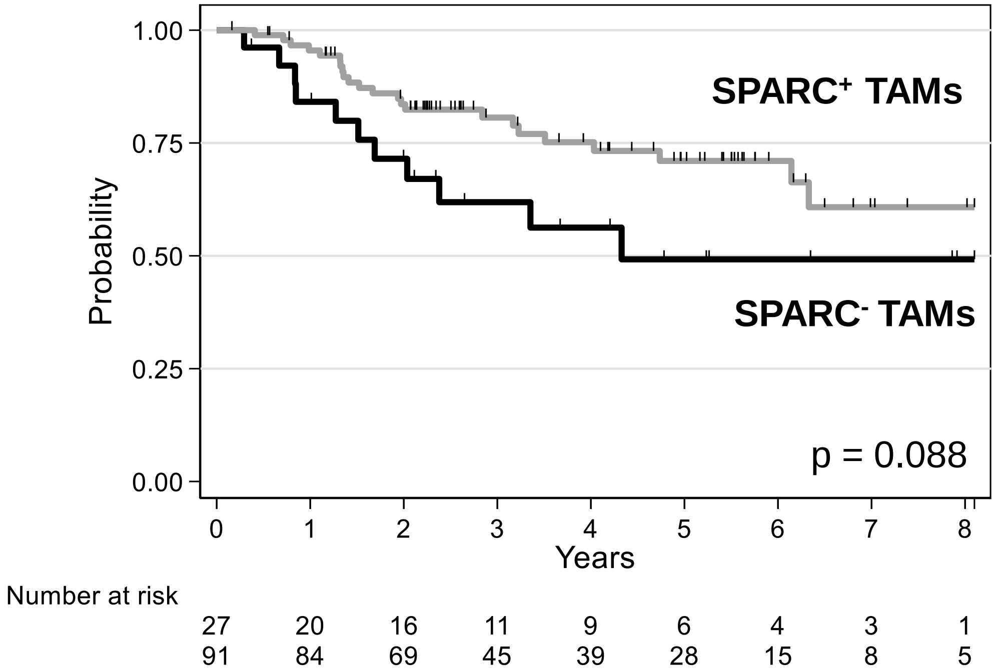

Figure S2. Relapse-free survival in function of SPARC status in TAMs.

Patients with TNBC were divided in two subgroups according to SPARC expression in TAMs: $SPARC^+$ and $SPARC^-$.

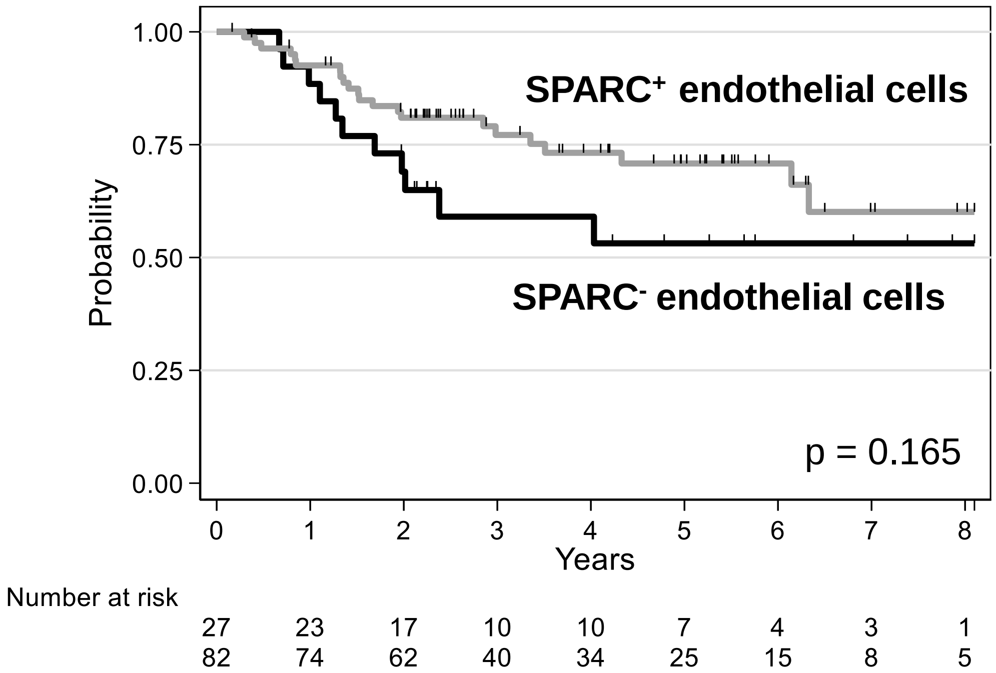

Figure S3. Relapse-free survival according in function of SPARC expression status in endothelial cells.

Patients with TNBC were divided in two subgroups according to SPARC expression in endothelial cells within the tumor microenvironment: $SPARC^+$ and $SPARC^-$.

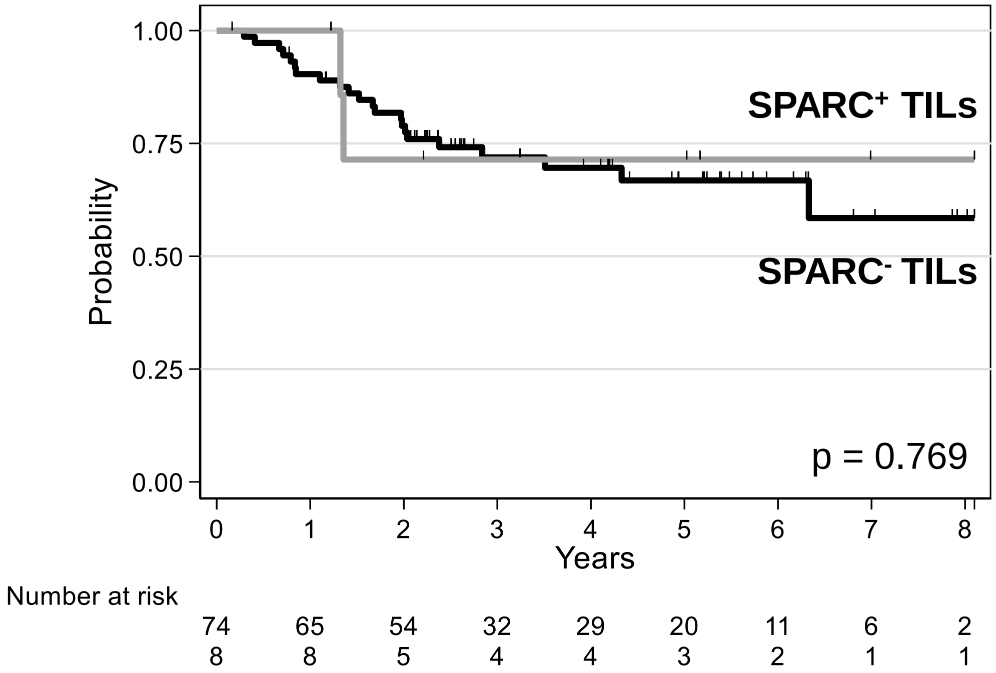

Figure S4. Relapse-free survival according to SPARC expression status in TILs. Patients with TNBC were divided in two subgroups according to SPARC expression in TILs: $SPARC^+$ and $SPARC^-$.

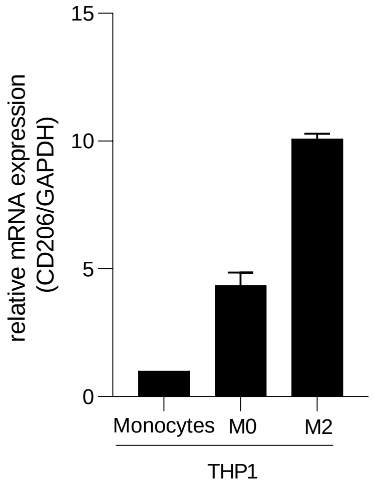

Figure S5. THP1 monocyte differentiation into M2 macrophages.

mRNA expression of the M2 macrophage marker CD206 was quantified by RT-qPCR in THP1 monocytes, M0-, and M2-polarized THP1 macrophages. Data were normalized to GAPDH expression level. Results are expressed as mean ± SD (n = 3).

SPARC	myCAFs	iCAFs	imPVL	dPVL	Myoepithelial	Endothelial
**myCAFs**	-	2.39e-63		1.07e-25	4.26e-59	3.19e-33
**iCAFs**	2.39e-63	-	2.22e-15	1.01e-51		5.48e-120
**imPVL**		2.22e-15	-		8.09e-22	
**dPVL**	1.07e-25	1.01e-51		-	9.12e-6	
**Myoepithelial**	4.26e-59		8.09e-22	9.12e-6	-	3.78e-24
**Endothelial**	3.19e-33	5.48e-120			3.78e-24	-

Figure S6. Differential *SPARC* gene expression in different cell populations within TNBC samples by single-cell RNA-seq data analysis.

The previously published single-cell RNA-seq dataset PRJEB35405 included five patients with TNBC[9]. P values were calculated with the Wilcoxon Rank Sum test and adjusted using the Bonferroni correction based on the total number of genes.

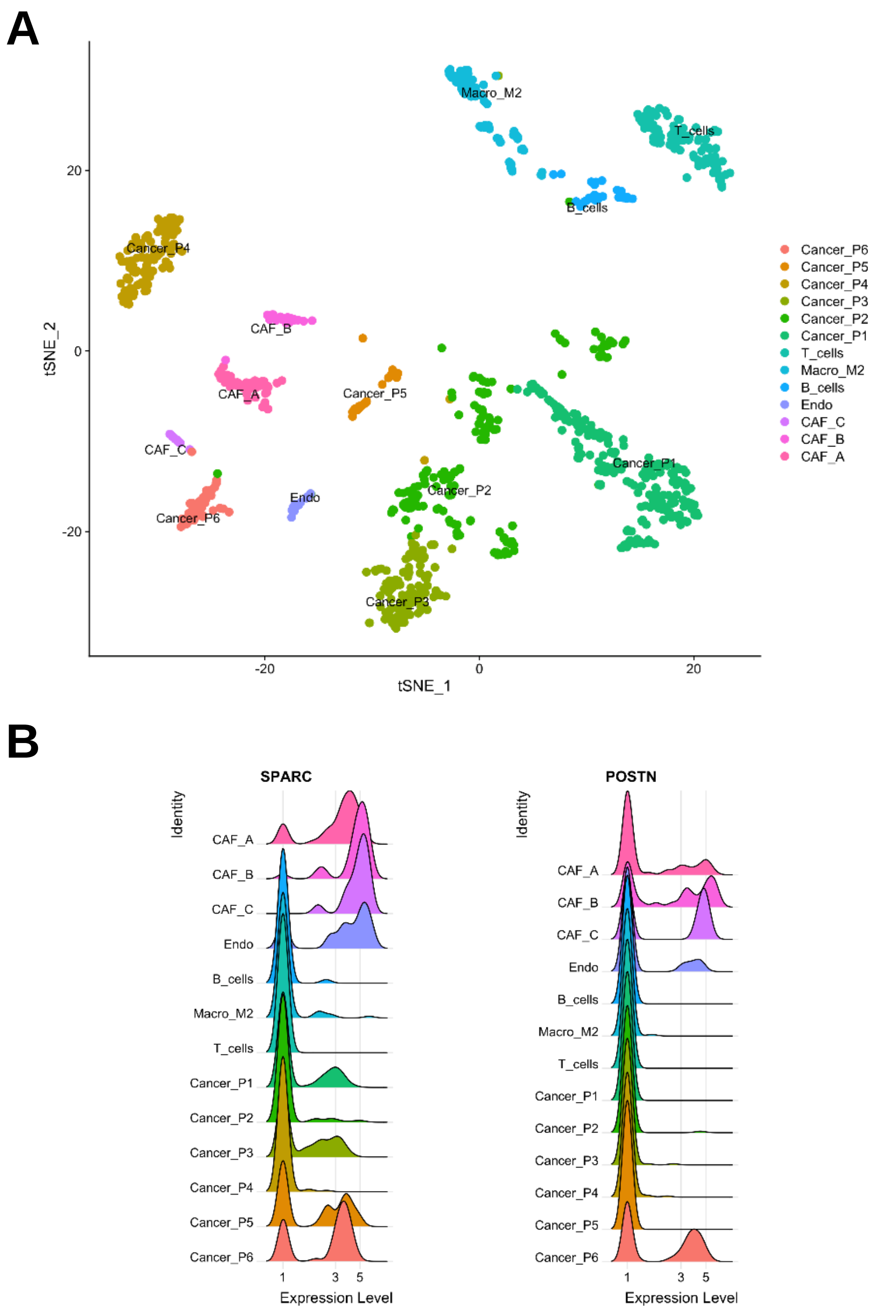

Figure S7. Expression of *SPARC* and *POSTN* mRNAs in TNBC by single-cell RNA-seq analysis.

(A) Cell populations. Thirteen cell populations were identified by single-cell RNA-seq analysis of the previously published GSE118390 dataset (n=6 TNBC samples), according to[10].

(B) *SPARC* and *POSTN* mRNA expression. *SPARC* and *POSTN* mRNA relative expressions in each of the 13 cell populations identified by single-cell RNA-seq analysis according to[10]. Individual cell populations were annotated as published in the original single-cell RNA-seq study[10] except for TNBC cells that are labelled Cancer_P1, _P2, _P3, _P4, _P5, _P6 (patient 1 to 6, respectively).

A

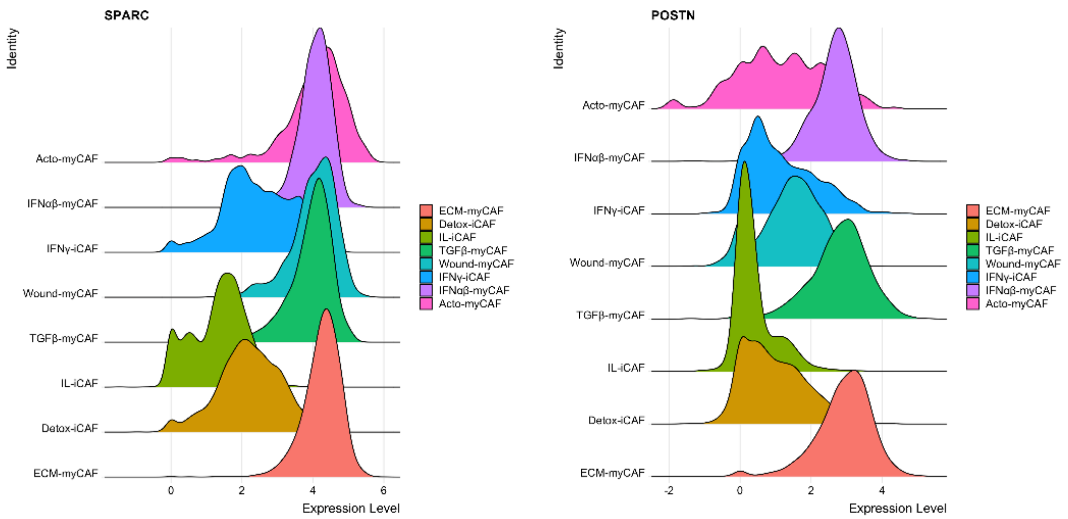

B

SPARC	Acto-myCAF	IFNαβ-myCAF	IFNγ-iCAF	Wound-myCAF	TGFβ-myCAF	IL-iCAF	Detox-iCAF	ECM-myCAF
Acto-myCAF	-		2.84e-76			1.47e-139	6.54e-116	
IFNαβ-myCAF		-	4.88e-173			0	0	
IFNγ-iCAF	2.84e-76	4.88e-173	-	1.26e-192	6.87e-222	5.86e-153		0
Wound-myCAF			1.26e-192	-		0	0	
TGFβ-myCAF			6.87e-222		-	0	0	
IL-iCAF	1.47e-139	0	5.86e-153	0	0	-	0	0
Detox-iCAF	6.54e-116	0		0	0	0	-	0
ECM-myCAF			0			0	0	-

Figure S8. Expression of *SPARC* and *POSTN* mRNAs in CAF-S1 clusters in breast cancer by single-cell RNA-seq analysis.

(A) Eight CAF-S1 clusters were identified in the previously published single-cell RNA-seq dataset EGAS00001004030 (n = 8 primary breast cancer samples) according to [11]. *SPARC* and *POSTN* mRNA relative expression in the eight CAF-S1 clusters, according to [11], is shown. Individual CAF-S1 clusters were annotated as published in the original single-cell RNA-seq study [11]. According to [11], myCAFs were identified in the following five clusters: ECM-myCAF: associated with extracellular matrix (ECM) remodeling, cell-substrate adhesion, and collagen formation; TGFb-myCAF: associated with TGFb signaling pathway and matrisome; Wound-myCAF: associated with assembly of collagen fibrils and wound healing; IFNab-myCAF: associated with IFNa/b signaling; Acto-myCAF: associated with the actomyosin complex. iCAF were identified in the following three clusters: Detox-iCAF: associated with the detoxification and inflammatory responses; IL-iCAF: associated with the response to growth factors, TNF signaling, and interleukin (IL) pathway; IFNg-iCAF: associated with the response to IFNg and cytokine-mediated signaling pathways.

(B) Differential *SPARC* gene expression analysis in the indicated cell populations; p values were calculated with the Wilcoxon Rank Sum test and adjusted using the Bonferroni correction based on the total number of genes.

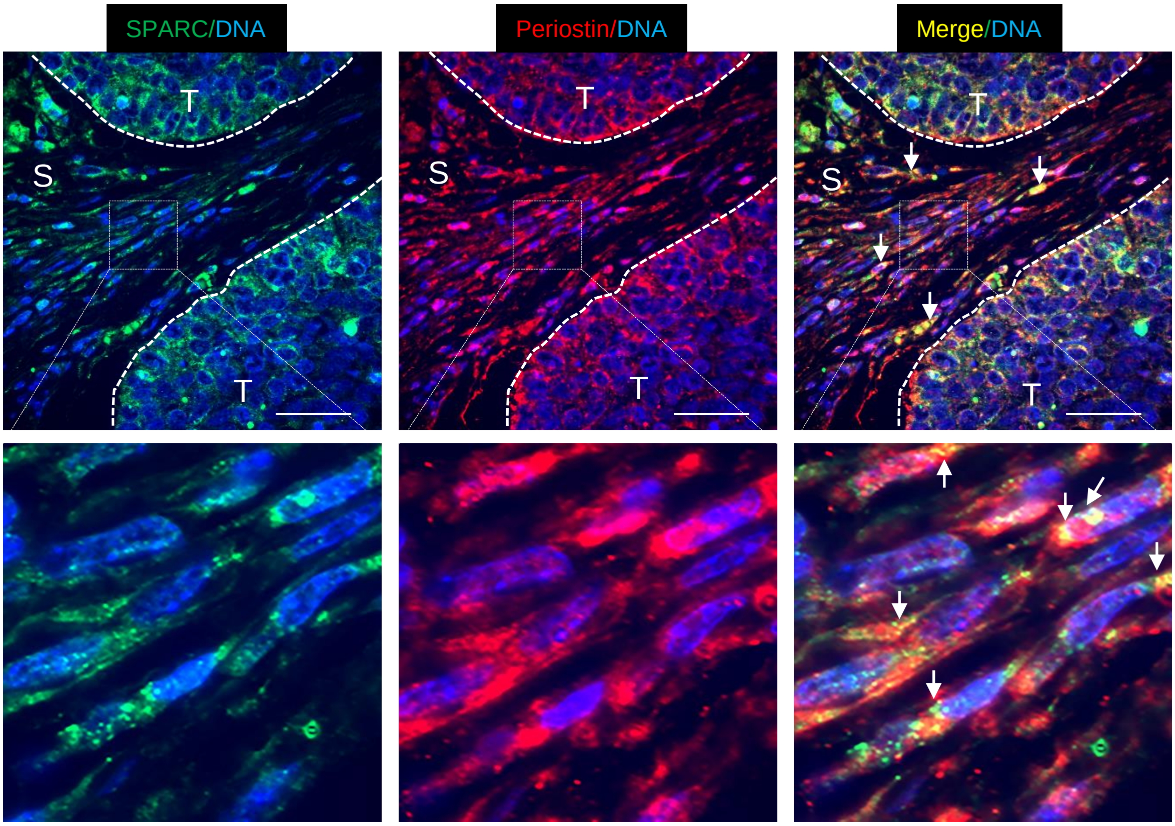

Figure S9. Co-localization of SPARC with periostin in TNBC PDX.

PDX B1995 tumor sections were co-incubated with an anti-SPARC polyclonal antibody (15274-1-AP) (green) and an anti-periostin monoclonal antibody (Proteintech) (red). Nuclei were stained with Hoechst 33342 (blue). Top panels: SPARC, periostin, and merge. Bottom panels: higher magnification of the boxed areas. Arrows indicate SPARC and periostin co-localization. Scale bar, 10 µm.

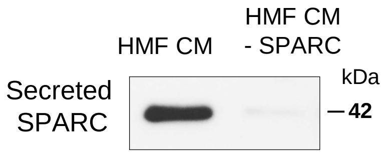

Figure S10. Immunodepletion of SPARC secreted in the conditioned medium from HMFs. SPARC was immunodepleted or not from HMF CM by immunoprecipitation. SPARC immunodepletion in HMF CM (HMF CM - SPARC) was confirmed by western blot analysis with an anti-SPARC monoclonal antibody (clone AON-5031).
